# Effects of dietary supplementation of a lipid-coated zinc oxide product on the fecal consistency, growth, and morphology of the intestinal mucosa of weanling pigs

**DOI:** 10.1186/s40781-017-0159-z

**Published:** 2018-01-30

**Authors:** Young-Jin Byun, Chul Young Lee, Myeong Hyeon Kim, Dae Yun Jung, Jeong Hee Han, Insurk Jang, Young Min Song, Byung-Chul Park

**Affiliations:** 1 Department of Animal Resources Technology, Gyeongnam National University of Science and Technology, Jinju, 52725 South Korea; 2 College of Veterinary Medicine and Institute of Veterinary Science, Kangwon National University, Chuncheon, 24341 South Korea; 3 Department of Animal Science and Biotechnology, Gyeongnam National University of Science and Technology, Jinju, 52725 South Korea; 4 Graduate School of International Agricultural Technology, Institute of Green Bio Science and Technology, Seoul National University, Pyeongchang, 25354 South Korea

**Keywords:** Weaned pig, Zinc, Growth, Diarrhea, Intestine

## Abstract

**Background:**

Dietary supplementation of zinc oxide (ZnO) to 2000 to 4000 mg/kg is known to be effective for the prevention and treatment of post-weaning diarrhea in the pig. Such a ‘pharmacological’ supplementation, however, can potentially result in environmental pollution of the heavy metal, because dietary ZnO is mostly excreted unabsorbed. Two experiments (Exp.) were performed in the present study to determine the effects of a lipid-coated ZnO supplement Shield Zn (SZ) compared with those of ZnO.

**Methods:**

In Exp. 1, a total of 240 21-day-old weanling pigs were fed a diet supplemented with 100 mg Zn/kg as ZnO (ZnO-100), ZnO-2500, SZ-100, or SZ-200 in 24 pens for 14 days on a farm with its post-weaning pigs exhibiting a low incidence of diarrhea. Exp. 2 was performed using 192 24-day-old piglets as in Exp. 1 on a different farm, which exhibited a high incidence of diarrhea.

**Results:**

In Exp. 1, fecal consistency (diarrhea) score (FCS) was less for the ZnO-2500 and SZ-200 groups than for the SZ-100 group (P < 0.05), with no difference between the SZ-100 and ZnO-100 groups. Both average daily gain (ADG) and gain:feed ratio were less for the SZ-200 group than for the ZnO-2500 group, with no difference between the ZnO-100 group and SZ-100 or SZ-200 group. The villus height (VH), crypt depth (CD), and VH:CD ratio of the intestinal mucosa were not influenced by the treatment. In Exp. 2, FCS was lowest for the ZnO-2500 group, with no difference among the other groups. However, neither the ADG nor gain:feed ratio was influenced by the treatment.

**Conclusion:**

Results suggest that physiological SZ supplementation has less beneficial effects than pharmacological ZnO for the alleviation of diarrhea irrespective of its severity and for promoting growth without influencing their integrity of the intestinal mucosal structures with little advantage over physiological ZnO in weanling pigs with a small pen size.

## Background

Weaning is accompanied by impaired structural integrity and physiological function of the intestinal villus due to the abrupt change in diet, which results in transient diarrhea and growth retardation in weanling pigs [1–3]. Dietary supplementation of zinc oxide (ZnO) to 2000 to 4000 mg/kg is known to be effective for the prevention and treatment of post-weaning diarrhea [4–6]; therefore, the first-phase post-weaning pig diet is supplemented with approximately 3000 mg ZnO/kg in many countries, including Korea [7, 8]. Such a pharmacological supplementation, however, can potentially result in environmental pollution of the heavy metal, because 75 to 90% of dietary ZnO is excreted unabsorbed [9, 10]. The EU therefore limits dietary Zn concentration to 150 mg/kg or less by legislation [11], which may lead non-European countries to adopting similar legislation. This has also called for the manufacture of high-bioactive ZnO additives that are just as effective at a dosage lower than the pharmacological ZnO, thereby enabling a reduction of supplemental Zn concentrations.

Shield Zn® (SZ) is a lipid-coated ZnO product that has been designed to maximize the delivery of the mineral particle to the intestine without being ionized in the stomach due to the lipid coating. The present group has reported that dietary provision of 72 mg Zn/kg using SZ was comparable to 2000 mg Zn/kg as ZnO in its effects on the alleviation of diarrhea and impaired weight gain and intestinal villus structure in weanling pigs challenged with enterotoxigenic *Escherichia coli* (ETEC) K88 [12, 13]. These effects of dietary SZ supplementation, however, were not consistent in unchallenged piglets in our previous studies. Supplementation of neither 100 to 250 mg Zn/kg as SZ nor 2500 mg Zn/kg as ZnO had any effect on the growth performance of the piglets which were housed in experimental-scale pens with a 4-animal unit size, as reported by Jang et al. [14]. In larger pens with a 34-piglet unit size [15], dietary provision of 100 mg Zn/kg as SZ enhanced the rate of weight gain, albeit to a lesser extent compared to that with 2500 mg Zn/kg as ZnO, without influencing the intestinal villus structure. The intestinal villus structure did not change due to 100 mg Zn/kg provided by SZ or 2500 mg Zn/kg by ZnO in either study, but an increase in villus height or the villus height:crypt depth ratio in response to 250 mg Zn/kg vs. 100 mg Zn/kg by SZ was detected in Jang et al. [14]. These results from the challenged and unchallenged piglets suggest that the effects of SZ in weanling pigs may vary depending on the ETEC infection status, pen size, and supplemented SZ concentration. The present study was therefore performed to determine the effects of ‘physiological’ dietary SZ compared with those of pharmacological as well as physiological ZnO in small pens in two groups of weanling pigs that had exhibited a low incidence and a high incidence of post-weaning diarrhea, respectively.

## Methods

Experiments (Exp.) 1 and 2 described below were conducted on two commercial swine farms with a low incidence and a high incidence of post-weaning diarrhea, respectively.

### Exp. 1

A total of 240 21-day-old weanling pigs with equal numbers of females and castrated males born to Duroc-sired Landrace × Yorkshire dams were allotted to twenty-four 1.3 m × 1.5 m pens according to the sex and body weight (low, medium, or high) under a randomized complete block design with 6 replicates (pens) per treatment and 10 piglets per pen. The piglets received a dietary provision of 100 mg Zn/kg as ZnO (ZnO-100), ZnO-2500, 100 mg Zn/kg as SZ (Shield Zn®; CTCBIO Inc., Seoul, Korea) with 10% (*w*/w) lipid coating (SZ-100), or SZ-200 supplemented to a basal diet (Table 1) which was formulated to meet the nutrient composition recommended by NIAS [16]. The animals were provided ad libitum with water and one of the four experimental diets for 14 days.

**Table 1:** Composition of the basal diet (as-fed basis)

Item	Content
Ingredient (%)	
Corn	33.16
Barley	8.00
Soybean meal	10.00
Dehulled soybean meal	10.00
Wheat bran	3.00
Limestone	0.30
Sweet whey	12.56
Lactose	4.20
Fish meal	5.00
Fermented soybean	4.15
Sucrose	3.00
Soy oil	3.00
Organic acids	0.70
Monocalcium phosphate	1.20
Salt	0.30
Vitamin premix^a)^	0.15
Mineral premix^b)^ (Zn-free)	0.20
Others^c)^	0.77
Total^d)^	99.69
Nutritional composition	
Digestible energy (Mcal/kg)	3.50
Crude protein (%)	18.50
Ether extract (%)	4.50
Lysine (%)	1.52

^a)^Provided per kg: 1500 IU vitamin A, 2000 IU vitamin D_3_, 65 IU vitamin E, 1.5 mg vitamin K, 1.0 mg thiamin, 6 mg riboflavin, 20 mg pantothenic acid, 25 mg niacin, 1.5 mg vitamin B_6_, 1 mg folic acid, 25 μg vitamin B_12_, 25 μg biotin, and 150 mg choline

^b)^Provided per kg: 160 mg Cu, 200 mg Fe, 40 mg Mn, 1 mg I, 0.15 mg Co, and 0.4 mg Se

^c)^Provided per total weight: 0.1% choline-HCl, 0.347% L-lysine-HCl (78%), 0.150% DL-methionine (99%), 0.114% L-threonine (99%), 0.009% L-tryptophan, and 0.05% ethoxyquin

^d)^Four experimental diets were supplemented to the basal diet with 125 and 3125 mg ZnO and with 139 and 278 mg of 10% lipid (*w*/w)-coated ZnO (Shield Zn®) per kg diet, respectively, as well as with 0 to 0.30% corn, to provide 100, 2500, 100, and 200 mg Zn/kg, respectively, and also to make 100.00 for the sum of percentages of all ingredients

Body weight was measured on days 0, 7, and 14. Average daily feed intake (ADFI) during each of days 0 to 7 and days 7 to 14 for each pen was calculated by dividing the total feed intake by the sum of the numbers of animals on feed. For the gain:feed ratio, the average daily gain (ADG) for each pen was calculated, followed by division of the calculated ADG by ADFI. Fecal consistency was scored subjectively as previously described [14, 15] except that this study used a 0-to-2 scale instead of the 1-to-3 scale used in the previous studies: 0, normal firm feces; 1, soft feces; and 2, diarrhea.

At the end of the feeding trial, a total of 24 animals randomly selected from each of the 24 pens were killed by electric stunning, after which the entire intestinal tracts were removed and the intestinal specimens were prepared as previously described [12, 17]. Briefly, a 5-cm cross-section excised from each of the duodenum, jejunum, and ileum was rinsed in phosphate buffered saline, fixed in 10% formalin, embedded in paraffin film, sliced using the microtome, mounted onto the slide, and stained with hematoxylin and eosin. The villus height (VH) and crypt depth (CD) of the intestinal mucosa were determined microscopically using the Diagnostic Insight visual analysis program (Olympus Co., Tokyo, Japan) and the average for each of the VH and CD measurements at four villi was taken as a replicate as also described [12, 17].

### Exp. 2

A total of 192 (Landrace × Yorkshire) × Duroc piglets were allotted to twenty-four 1.4 m × 1.5 m pens at weaning at 24 days of age, with 6 replicates (pens) per treatment and 4 females and 4 castrated males per pen. The animals were subjected to the same 14-day feeding trial as in Exp.1, including the scoring of fecal consistency and the measurement and calculation of growth performance, but histological examination was not included for Exp. 2.

### Statistical analysis

Data were analyzed as a randomized complete block design using the General Linear Model procedure of SAS (SAS Inst. Inc., Cary, NC, USA) for all the variables in Exp. 1 and for all variables except the ADFI and gain:feed ratio in Exp. 2. The pen, which was the experimental unit for both Exp. 1 and 2, was used as the error term to test the effect of the treatment. In the analysis of the fecal consistency score (FCS), the effects of the day and the day × treatment interaction were tested using the day × pen as the error term. The means were separated by *t* test at the 5% significance level.

## Results

### Exp. 1

The FCS on day 0, which was almost zero indicating normal firm feces, did not differ among the experimental groups (Table 2). The FCS increased (*P* < 0.05) between days 0 and 7 and returned to day 0 levels at the end of the experiment on day 14 for all groups except ZnO-2500, which remained at day 0 levels on days 7 and 14. The FCS on day 7 was less for the ZnO-2500 group than for the ZnO-100 and SZ-100 groups as well as for the SZ-200 group vs. the SZ-100 group. Overall, the mean FCS was less for the ZnO-2500 and SZ-200 groups than for the SZ-100 group, and no difference was seen between the ZnO-100 and SZ-100 groups or between the ZnO-2500 and SZ-200 groups.

**Table 2:** Effects of dietary supplementation of the lipid-coated ZnO Shield Zn® (SZ) vs. ZnO on fecal consistency and growth performance in weanling pigs (Exp. 1)

Item	ZnO (mg Zn/kg)		SZ (mg Zn/kg)		SEM	*P* value
	100	2500	100	200		
FCS^1),2)^						
Day 0	0.07^y^	0.03	0.03^y^	0.00^y^		
Day 7	0.52^b,x^	0.25^c^	0.93^a,x^	0.43^bc,x^	0.08^3)^	
Day 14	0.24^y^	0.07	0.21^y^	0.15^y^		
Overall^4)^	0.28^ab^	0.12^c^	0.39^a^	0.19^bc^	0.04	0.002
Body weight^1)^ (kg)						
Day 0	6.10	6.36	6.30	6.45	0.11	0.197
Day 7	7.70	7.87	7.76	7.69	0.12	0.712
Day 14	8.92^b^	9.70^a^	9.27^ab^	8.70^b^	0.22	0.043
ADG^1)^ (g)						
Days 0 to 7	229^a^	216^a^	207^a^	176^b^	9	0.009
Days 7 to 14	165^b^	262^a^	218^ab^	150^b^	22	0.013
Overall	197^bc^	241^a^	212^ab^	155^c^	14	0.011
ADFI^5)^ (g)						
Days 0 to 7	261	257	253	249	8	0.792
Days 7 to 14	372	456	387	355	28	0.122
Overall	316	357	320	302	17	0.201
Gain:feed^5)^						
Days 0 to 7	0.88	0.84	0.82	0.76	0.03	0.062
Days 7 to 14	0.43^c^	0.58^a^	0.55^ab^	0.44^bc^	0.03	0.015
Overall	0.62^bc^	0.68^a^	0.66^ab^	0.56^c^	0.02	0.010

*Exp.* experiment, *SEM* standard error of mean, *FCS* fecal consistency score, *ADG* average daily gain, *ADFI* average daily feed intake

^a-c^Means with no common superscript within a row differ (P < 0.05)

^x,y^Means with no common superscript within a column differ (P < 0.05)

^1)^Data are least squares means of 6 pens, with 10 piglets per pen

^2)^Scored subjectively: 0, normal firm feces; 1, soft feces; 2, diarrhea

^3)^Applies to all day × treatment combinations

^4)^The *P* values for the day and day × treatment were <0.001 and 0.009, respectively

^5)^Data are least squares means of 6 pens

The ADG during the first 7 days was smallest for the SZ-200 group and was not different among the rest of the experimental groups. The ADG during the second 7 days was less for the SZ-200 group than for the ZnO-2500 group, with no difference among the SZ-100 and -200 and ZnO-100 groups. Overall, ADG for the entire 14 days was greater for the ZnO-2500 group than for the SZ-200 and ZnO-100 groups as well as for the SZ-100 group vs. SZ-200, not being different between the SZ-100 group and either of the two ZnO groups.

The ADFI was not affected by the treatment during the period of days 0–7, 7–14, or 0–14. The gain:feed ratio was not influenced by the treatment during the first 7-day period. The gain:feed ratio during the second 7-day period was greater for the ZnO-2500 and SZ-100 groups than for the ZnO-100 group. Furthermore, the gain:feed ratio for entire 14-day period was greater for the ZnO-2500 and SZ-100 groups vs. SZ-200 group, with no difference between the SZ-100 group and either of the ZnO-100 and ZnO-2500 groups.

None of the effects of the treatment on VH, CD, and VH:CD ratio in the duodenum, jejunum, and ileum was significant (*P* > 0.05; Table 3).

**Table 3:** Effects of dietary supplementation of the lipid-coated ZnO Shield Zn® (SZ) vs. ZnO on intestinal mucosal morphology of weanling pigs (Exp. 1)

Item	ZnO (mg Zn/kg)		SZ (mg Zn/kg)		SEM	P value
	100	2500	100	200		
Duodenum^a)^						
VH (μm)	290	293	271	244	19	0.260
CD (μm)	216	236	208	226	13	0.504
VH:CD ratio	1.35	1.27	1.34	1.07	0.11	0.261
Jejunum^a)^						
VH (μm)	297	280	276	245	16	0.187
CD (μm)	209	234	206	205	12	0.685
VH:CD ratio	1.43	1.28	1.37	1.22	0.11	0.540
Ileum^a)^						
VH (μm)	234	223	257	224	10	0.089
CD(μm)	171	182	176	181	14	0.947
VH:CD ratio	1.39	1.27	1.48	1.28	0.08	0.223

*Exp*. experiment, *SEM* standard error of mean, *VH* villus height, *CD* crypt depth

^a)^Data are means of 6 pens, with one animal analyzed per pen

### Exp. 2

The FCS increased between days 0 and 7 and decreased between days 7 and 14 as in Exp. 1 (Table 4). The FCS on day 14 was an intermediate value between those on days 0 and 7 in the ZnO-100 and SZ-100 groups whereas it did not differ from that on day 0 in the ZnO-2500 and SZ-200 groups. The mean FCS was greater for the SZ-100 and SZ-200 groups than for the ZnO-2500 group, but was not different between the ZnO-100 group and either of the two SZ groups or between the SZ-200 and SZ-100 groups.

**Table 4:** Effects of dietary supplementation of the lipid-coated ZnO Shield Zn® (SZ) vs. ZnO on fecal consistency and growth performance of weanling pigs (Exp. 2)

Item	ZnO (mg Zn/kg)		SZ (mg Zn/kg)		SEM	*P* value
	100	2500	100	200		
FCS^1),2)^						
Day 0	0.11^z^	0.02^y^	0.04^z^	0.23^y^		
Day 7	0.84^ab,x^	0.63^b,x^	1.02^a,x^	0.84^ab,x^	0.10^3)^	
Day 14	0.50^y^	0.29^y^	0.36^y^	0.52^y^		
Overall^4)^	0.47^a^	0.31^b^	0.47^a^	0.53^a^	0.05	0.035
Body weight^1)^ (kg)						
Day 0	6.86	6.82	6.70	6.81	0.15	0.899
Day 7	7.56	7.67	7.15	7.17	0.15	0.056
Day 14	9.41	9.89	9.18	9.26	0.24	0.189
ADG^1)^ (g)						
Days 0 to 7	103^ab^	131^a^	63^b^	60^b^	18	0.035
Days 7 to 14	257	317	282	274	16	0.097
Overall	180	220	174	168	14	0.065
ADFI^5)^ (g)						
Days 0 to 7	182	171	154	154	10	0.203
Days 7 to 14	313	327	361	312	18	0.242
Overall	247	249	253	230	11	0.503
Gain:feed^5)^						
Days 0 to 7	0.54^ab^	0.71^a^	0.36^b^	0.27^b^	0.10	0.037
Days 7 to 14	0.90	1.00	0.80	0.91	0.06	0.180
Overall	0.76	0.88	0.70	0.77	0.06	0.185

*Exp.* experiment, *SEM* standard error of mean, *FCS* fecal consistency score, *ADG* average daily gain, *ADFI* average daily feed intake

^a,b^Means with no common superscript within a row differ (*P* < 0.05)

^x-z^Means with no common superscript within a column differ (*P* < 0.05)

^1)^Data are least squares means of 6 pens, with 8 piglets per pen

^2)^Scored subjectively: 0, normal firm feces; 1, soft feces; 2, diarrhea

^3)^Applies to all day × treatment combinations

^4)^The P values for the day and day × treatment were <0.001 and 0.447, respectively

^5)^Data are least squares means of 6 pens

The ADG during the first 7 days was less for the SZ-100 and -200 groups than for the ZnO-2500 groups. However, the treatment effects on ADG during the second 7 days and for the entire 14 days were not significant.

The ADFI did not differ among the treatments during any experimental period. The gain:feed ratio during days 0–7 was less for the SZ-100 and SZ-200 groups than for the ZnO-2500 group; however, the gain:feed ratio was not influenced by the treatment during the period of days 7–14 or 0–14.

When the data from Exp. 1 and Exp. 2 were pooled, the FCS was greater for Exp. 2 than for Exp. 1 (0.24 vs. 0.45; SEM = 0.03; *P* < 0.01) as expected. Moreover, the ADG and gain:feed ratio during the first 7 days were greater (P < 0.01) for Exp. 1 (206 ± 7 g and 0.82 ± 0.06, respectively) than for Exp. 2 (92 ± 7 g and 0.47 ± 0.06, respectively). During the second 7 days, however, both ADG and gain:feed ratio were greater (P < 0.01) for Exp. 2 (283 ± 9 g and 0.90 ± 0.04, respectively) vs. Exp. 1 (200 ± 8 g and 0.48 ± 0.04, respectively).

## Discussion

The FCS was visibly greater in Exp. 2 than in Exp. 1, as was expected, indicating a greater severity of post-weaning pig diarrhea in the former. Moreover, FCS of the weanling pigs was lower due to the ZnO-2500 treatment vs. ZnO-100 for both Exp. 1 and 2, matching published results from previous studies [4, 18] including Park et al. [15], whose feeding trial was performed at the same farm where Exp. 1 was performed. In addition, the lack of effect on FCS for SZ-100 vs. ZnO-100 was also consistent with our previous results with commercial piglets [15].

The lesser FCS in response to SZ-200 vs. SZ-100 only in Exp. 1 suggests that the dose-response effect of SZ on FCS may depend on the severity of post-weaning diarrhea, yet further studies are necessary to confirm this possibility. With respect to the dose effect of the lipid-coated ZnO, Shen et al. [19] have reported that FCS of the post-weaning pigs was lowered by dietary supplementation with 380 or 570 mg Zn/kg, but not with a lower or higher concentration up to 1140 mg Zn/kg, provided by a lipid-coated ZnO product compared to that with 250 mg Zn/kg provided by ZnO. In Wang et al. [20], post-weaning pig diarrhea was alleviated by provision of 1500 mg Zn/kg with another lipid-coated ZnO product to the same extent elicited by 3000 mg Zn/kg as ZnO, but the dose effects for the former were not examined in this study. These results, however, cannot be directly compared with those of the present study, because how the ZnO particle was coated and consequently what percentage of the mineral particle was bio-available in the intestine are unknown for each of the lipid-coated ZnO products.

It was noteworthy that the ADG and gain:feed ratio during days 0–7 were much less in Exp. 2 than in Exp. 1, but these performance variables during days 7–14 were much greater in Exp. 2. This suggests that the growth of the piglets was dampened due to the high severity of post-weaning diarrhea during the first week in Exp. 2, whereas a compensatory growth occurred during the second week with the decreased severity of diarrhea between days 7 and 14. Regarding the effects of the treatment on performance, the greater ADG and greater gain:feed ratio for the ZnO-2500 treatment vs. ZnO-100 in Exp. 1 were consistent with the known growth-promoting effect of pharmacological ZnO published in the literature [4, 21] including the report of our own [15]. However, the lack of effect on ADG for the SZ-100 treatment vs. ZnO-100 in both Exp. 1 and 2 was inconsistent with the low, but significant growth-enhancing effect for the former observed in the previous study [15], and the dose effects of SZ on the ADG and gain:feed ratio were not consistent between Exp. 1 and 2.

The VH and VH:CD ratio are commonly used as positive indices for estimating the integrity of the intestinal mucosal structure, whereas CD is regarded as a negative index [6, 22]. The VH and VH:CD ratio do not change consistently in response to pharmacological ZnO, being increased [23], unchanged [24], or even decreased [25] by the dietary ZnO treatment. Reasons for this inconsistent response of the piglets to dietary ZnO are not clearly known, but are apparently associated with the diarrhea-reducing effect and antimicrobial properties of ZnO [6, 26, 27]. In this context, amelioration of impaired intestinal villus structure caused by an ETEC K88 challenge by the ZnO or SZ treatment in weanling pigs was associated with reduced intestinal adhesion and fecal shedding of the challenged pathogen accompanied by reduced FCS in our previous studies [12, 13]. By analogy, the lack of effects of either ZnO-2500 or SZ treatment on the structural variables in the present study was probably because the presumptively impaired intestinal villus structure due to weaning recovered to a normal state after several days post-weaning, which was extrapolated from the transient increase and subsequent decrease of FCS by day 14 when the intestinal tissues were sampled.

## Conclusions

The results of the present study indicated that pharmacological ZnO, but not physiological SZ, was effective for the alleviation of diarrhea irrespective of its severity and for promoting growth without influencing their integrity of the intestinal mucosal structures in post-weaning pigs when they were housed in small unit-size pens of 10 animals or less. However, relative effects for the SZ-100 and SZ-200 treatments on post-weaning diarrhea and growth performance were inconclusive. Obviously, more studies are necessary to determine the comprehensive dose-response effects for SZ, because only two doses of SZ were included in the present study to examine the relative effects of SZ near the upper limit of the in-feed Zn concentration of the EU (150 mg/kg [11]).

## Abbreviations

ADFI: average daily feed intake
ADG: average daily gain
CD: crypt depth
ETEC: enterotoxigenic Escherichia coli
FCS: fecal consistency score
SZ: Shield Zn®
VH: villus height
