# Correction to: effects of dietary supplementation of a lipid-coated zinc oxide product on the fecal consistency, growth, and morphology of the intestinal mucosa of weanling pigs

**DOI:** 10.1186/s40781-018-0161-0

**Published:** 2018-03-05

**Authors:** Young-Jin Byun, Chul Young Lee, Myeong Hyeon Kim, Dae Yun Jung, Jeong Hee Han, Insurk Jang, Young Min Song, Byung-Chul Park

**Affiliations:** 1 Department of Animal Resources Technology, Gyeongnam National University of Science and Technology, Jinju, 52725 South Korea; 2 College of Veterinary Medicine and Institute of Veterinary Science, Kangwon National University, Chuncheon, 24341 South Korea; 3 Department of Animal Science and Biotechnology, Gyeongnam National University of Science and Technology, Jinju, 52725 South Korea; 4 Graduate School of International Agricultural Technology, Institute of Green Bio Science and Technology, Seoul National University, Pyeongchang, 25354 South Korea

## Correction

Due to a technical issue this article [1] was accidentally published in volume 59, the correct volume for this article is volume 60.
